# Combined Soil Microorganism Amendments and Foliar Micronutrient Nanofertilization Increased the Production of *Allium cepa* L. through Aquaporin Gene Regulation

**DOI:** 10.3390/life14010004

**Published:** 2023-12-19

**Authors:** José A. Berna-Sicilia, Mercy Quizhpe-Romero, María Hurtado-Navarro, José A. Pascual, Micaela Carvajal, Gloria Bárzana

**Affiliations:** 1 Aquaporins Group, Plant Nutrition Department, Centro de Edafología y Biología Aplicada del Segura (CEBAS-CSIC), Campus Universitario de Espinardo, Edificio 25, 30100 Murcia, Spain; joseangel.berna@edu.upct.es (J.A.B.-S.); mercyelizabeth.quizhper@um.es (M.Q.-R.); mhnavarro@cebas.csic.es (M.H.-N.); 2 Enzymology and Bioremediation of Soils and Organic Waste Department, Centro de Edafología y Biología Aplicada del Segura (CEBAS-CSIC), Campus Universitario de Espinardo, Edificio 25, 30100 Murcia, Spain; jpascual@cebas.csic.es

**Keywords:** *Allium cepa* L., nanoparticles, soil amendments, microorganisms, precision agriculture, aquaporins

## Abstract

The aim of this study was to investigate the impact of changes in aquaporin expression on the growth of onion (*Allium cepa* L.) plants when subjected to dual applications of microorganism-based soil amendments and foliar nanoencapsulated mineral nutrients. Multiple physiological parameters related to water, gas exchange, and nutrient content in leaf, root, and bulb tissues were determined. Additionally, the gene expression of aquaporins, specifically PIP1, PIP2 (aquaporin subfamily plasma membrane intrinsic protein), and TIP2 (aquaporin subfamily tonoplast intrinsic protein), was analyzed. The findings revealed that the foliar application of nutrients in a nanoencapsulated form significantly enhanced nutrient penetration, mobilization, and overall plant growth to a greater extent than free-form fertilizers. Amendments with microorganisms alone did not promote growth but influenced the production of secondary metabolites in the bulbs. The combination of microorganisms and nanoencapsulated mineral nutrients demonstrated synergistic effects, increasing dry matter, mineral content, and aquaporin gene expression. This suggests that aquaporins play a pivotal role in the transport of nutrients from leaves to storage organs, resulting in the overexpression of PIP2 aquaporins in bulbs, improved water uptake, and enhanced cell growth. Therefore, the combined treatment with microorganisms and nanoencapsulated mineral nutrients may be an optimal approach for enhancing onion productivity by regulating aquaporins under field conditions.

## 1. Introduction

Several factors affect the low fertility of soil, including the excessive overexploitation of resources and the toxic accumulations of many chemicals due to the indiscriminate use of fertilizers and pesticides [1]. All of these give rise to imbalances in the rhizosphere, affecting the soil–plant system, which becomes more susceptible to environmental stress, thereby compromising the survival of plants and the resilience of crops in the context of climate change. Onion (*Allium cepa* L.) is one of the most cultivated vegetables in the world [2] due to its well-known nutritional, culinary, and medicinal properties. However, the low development of secondary roots makes this crop very vulnerable to changes in soil–plant systems [3].

The microorganisms involved in the rhizosphere of plants, known as rhizobiome, are important in the way in which nutrients pass through the root system and intervene in their assimilation capacity, depending on their presence and abundance [4,5]. Under natural conditions, the rhizosphere promotes the selection of healthy microorganisms, favoring the optimization of existing natural resources [6]. However, this natural selection is often disrupted in soils intended for agriculture, due to the use of chemicals that interfere with rhizosphere microbiome relationships. Consequently, it has been demonstrated that the re-establishment of a healthy rhizobiome should be a priority in agriculture [7]. Amendments with microorganisms have shown their effectiveness in soil reconstitution [8]; their success necessitates a complementary commitment to reducing some other destabilizing factors. In this sense, reducing chemical fertilizers and pesticides should be a key objective, as it is essential for establishing a stable, long-lasting, sustainable, and resilient soil–plant system [6].

To achieve this goal, nanomaterials applied as fertilizers to soil–plant systems have garnered special interest [9]. Several nanoparticles, such as carbon nanotubes, or metals such as Au, Ag, and metal oxides, have been described as having excellent properties for plants, as they have been shown to enhance plant growth and development and also play an essential role in stress amelioration [10]. However, these nanoparticles (NPs) have also been recalled due to some toxicity risks, causing negative effects on growth, physiology, and yield [11,12]. According to the potential toxicity, new types of nanoencapsulations have been used due to their characteristics as hydrophobic vesicles obtained from biological materials. Nanoencapsulations from agricultural by-products have been tested in different crops, with interesting results observed in terms of bioavailability, compatibility, and efficiency as nutrient nanocarriers [13,14,15], without generating waste or leading to the accumulation of toxic elements in the ecosystem. In this way, the biodegradability of the coating reduces environmental impact and any risk of toxicity.

Aquaporins are intrinsic membrane proteins responsible for the movement of water and solutes into cells in all living organisms. In plants, there are many different isoforms with specific functions, and their relationships to crop growth and tolerance to stress have been studied widely. In that sense, aquaporins are fundamental in the physiological responses of plants due to their involvement in nutrient and water absorption and accumulation, directly influencing the main processes of nutrition, photosynthesis, and growth, and are essential to plants in terms of coping with environmental changes [16]. In onion cultivars, a relationship between aquaporins from the TIP and PIP subgroups (tonoplast intrinsic proteins and plasma membrane intrinsic Protein, respectively) and tolerance range to abiotic stress has been found to be related to micronutrient availability [17]. Given the essentiality of those proteins in the optimization of existing natural resources, their regulation should occur according to the availability of water and nutrients, which ultimately depends on soil availability and structure. On the other hand, the application of foliar fertilization has been proven to modify the expression of aquaporins at the whole-plant level, and its regulation seemed to be essential in improving water and nutrient immobilization when micronutrients were applied using nanovesicles [15].

In this way, the aim of this study was to determine if the addition of microorganisms to soil and the application of foliar mineral nutrient nanofertilizers produced a beneficial effect on the primary or secondary metabolism of onion plants. Additionally, whether or not the expression of aquaporins may be involved in this response was also explored. To test this hypothesis, micronutrients in free or nanoencapsulated formats were applied alone and in combination with a liquid organic material containing a pool of microorganisms. Many physiological parameters related to water, gas exchange, and nutrient content in the leaves, roots, and bulb tissues were measured, and the gene expressions of PIP1, PIP2, and TIP2 aquaporins were analyzed in relation to *Allium cepa* L.

## 2. Materials and Methods

### 2.1. Experimental Design and Growth Conditions

The onion plants, variety A-15 from the Javaloyes company (Cox, Alicante, Spain), were grown following the standard procedures in onion production fields located in the southeast of Spain. The mean temperatures during the experiment were a maximum of 15 °C and a minimum of 4 °C in January and 22 °C as the maximum and 12 °C as the minimum in May. The average humidity was 64%. The plants were drip-irrigated with fertigation using CaNO_3_ (7.5 g m^−2^) and K_2_NO_3_ (5.2 g m^−2^). Onion seedlings were transplanted in the middle of January into the soil in 200 m long rows, with a distance of 1 m between the rows. The spacing between the onions was 0.2 m. Fresh onion samples were collected at the end of the crop cycle when they were ready for the market at the beginning of May. Two random blocks were selected that included 6 planting rows, and each one received one treatment. A split plot design was applied for the experiment. At each plot, 3 subsectors that included the 6 treatments were chosen for plant sampling. Therefore, for each treatment, 18 plants were collected, corresponding to 9 per sector and 3 plants per subsector.

The foliar application was a mixture of Fe 3.2% *w*/*v*, Cu 0.2% *w*/*v* Mn 2.9% *w*/*v*, Zn 0.7% *w*/*v*, B 0.8% *w*/*v*, and Mo 0.04% *w*/*v* (Microfol, NUFOL, S.L. Granada, Spain) without encapsulation and nanoencapsulated in vesicles (0.1% with vesicles according to Rios et al. [15]). The root application of the fungi *Trichoderma harzianum* Rifai strain T78 (Trichosym SYMBORG Ltd., Murcia, Spain) in combination with the mixture of bacteria *Azospirillum brasilense* Tarrand, Krieg & Döbereiner, *Acinobacter jhonsonii* Brisou & Prévot, *Acetobacter fabarum* Cleenwerck, *Nocardiopsis alba* Kroppenstedt, *Candida boidinii* Ramírez-Gómez, and *Penicillium chrysogenum* Thom (Vitasoil, SYMBORG Ltd. Murcia, Spain) had a total final dose of 4 L per hectare, which was provided to the rhizosphere through the drip irrigation system with an isolated pump. The six different treatments were: (I) control, consisting of water for the foliar application, plus the usual fertilization through the drip irrigation system as the entire experiment (C); (II) foliar fertilizer application (FF); (III) foliar fertilizer nanoencapsulated application (EF); (IV) microbial root application (M); (V) a combination of foliar fertilizer plus the microbial root application (FF + M); and (VI) a combination of the nanoencapsulated foliar fertilizer plus the microbial root application (EF + M). The foliar treatments were applied two times, 13 days and 3 days before harvest, according to previous experiments performed to determine the most efficient application times in terms of nutrient penetrability. The microbial root treatments were applied six times throughout the growth period: two times during the first month, and once a month until harvest, with the last application being 13 days before harvest, coinciding with the first foliar treatment.

### 2.2. Fresh and Dry Weight

Samples were collected and separated into roots, bulbs, and leaves in the field. They were immediately weighted (FW) in a KERN PCB 3500-2 scale balance (Balingen, Germany). Additionally, the diameter of the bulbs was measured in fresh bulbs with a Digital Caliper 0150 mm (Bel-Art Products, Pequannock, NJ, USA). Dry weight (DW) was obtained by drying the sample in an oven (Dry-Big, J.P. Selecta. Barcelona, Spain) at 75 °C for 48 h until a constant weight was reached.

### 2.3. Leaf, Root, and Bulb Osmotic Potential

The osmotic potential was measured, differentiating between leaves, roots, and bulbs. Samples were frozen at −20 °C in a freezer (Ignis CO470 EG, Whirlpool Corporation., Benton Harbor, MI, USA). They were subsequently thawed, pressed, and centrifuged (10,000× *g*) in a refrigerated benchtop centrifuge (Hettich Universal 320 R, Kirchlengern, Germany) to obtain the cell extract. The osmotic potential of the leaf, root, and bulb extracts was calculated after measuring extract osmolarity using a freezing-point depression osmometer (Digital Osmometer, Roebling, Berlin, Germany).

### 2.4. Gas Exchange

Gas exchange parameters, such as transpiration, stomatal conductance, assimilation rate, and internal CO_2_, were measured in five fully developed leaves using a TPS-2 Portable Photosynthesis System gas exchange meter (PP Systems, Inc., Amesbury, MA, USA). Measurements were taken when the stomata were open, corresponding to the period from 09:00 a.m. to 11:00 a.m.

### 2.5. Ion Analysis

All the dried samples, differentiating between roots, leaves, and bulbs, were ground into a fine powder in a mill grinder (model A10, IKA, Staufen, Germany). The macro and micro mineral contents were analyzed using Inductively Coupled Plasma-Optical Emission Spectrometry (ICP-OES) on a Thermo ICAP 6500 Duo instrument (Thermo Fisher Scientific, Waltham, MA, USA). Leaves, bulbs, and roots were collected, dried, and ground into a fine powder, and a total of 200 mg of each sample were added to a 25 mL tube along with a mixture of 4 mL of 68% HNO_3_ and 1 mL of 33% H_2_O_2_ for digestion. Additionally, a Teflon reactor contained 300 mL of high-purity deionized water, 30 mL of 33% H_2_O_2_, and 2 mL of 98% H_2_SO_4_. The microwave heating digestion program consisted of three steps: starting at 20 °C and 40 bar, increasing by 10 bar per minute for 30 min until reaching 220 °C, and maintaining the temperature at 220 °C for 20 min. After cooling, the mineralized samples were transferred to 10 mL (for micro minerals) and 25 mL (for macro minerals) double-gauge tubes, and the volume was adjusted using high-purity deionized water. Calibration standards were prepared using a multi-mineral standard solution containing 31 minerals supplied by SCP Science (Baie-D′Urfe, QC, Canada) in high-purity deionized water. ICP-OES analyses included two control samples of high-purity deionized water and a multi-mineral standard. The mineral concentrations were calculated using the formula:

$$\frac{mg}{kg}=\frac{\left(C\times D\right)}{W}$$

where *C* represents the mineral concentration, *D* is the dilution factor, and *W* is the sample weight. These analyses were conducted at the Ionomics Laboratory (CEBAS-CSIC).

### 2.6. Determination of Soluble Sugars

Bulbs (10 g) were homogenized using a Polytron homogenizer (Kinematica PT 2500E, Luzern, Switzerland) after adding the equivalent amount of deionized water. The substance obtained was filtered through Miracloth 475855 (Millipore, Burlington, MA, USA) and centrifuged at 4000× *g* in a refrigerated benchtop centrifuge (Hettich Universal 320 R, Kirchlengern, Germany) to obtain a clear supernatant. The soluble sugars (SS) were determined according to the method described by Yemm and Willis [18]: 10 µL samples of juice from the bulbs were taken and diluted with 990 µL of demineralized water. After this, extract aliquots of 50 µL were taken and mixed with 950 µL of pure water and 2 mL of anthrone reagent (2 g L^−1^ of H_2_SO_4_). The mix was heated in a water bath (Fisherbrand, Watertown, MA, USA) at 100 °C for 8 min and cooled in an ice bath to stop the reaction. The determinations were performed by colorimetry in a spectrophotometer (Helios Gamma, Unicam Limited, Cambridge, UK) by recording the readings at a wavelength of 620 nm. The SS content was calculated from a standard sucrose curve that ranged from 0 to 100 mg mL^−1^.

### 2.7. Pungency and Total Phenolic Concentration

The amount of pyruvic acid, which is the commonly accepted measure of onion pungency, was determined in the different treatments according to the method described by Anthon and Barrett [19]. Fresh onions were sliced after removing the outer skin and ends. Samples (10 g) were homogenized with a Polytron homogenizer (Kinematica PT 2500E, Luzern, Switzerland) after adding the equivalent amount of deionized water. The substance obtained was filtered through Miracloth 475855 (Millipore, Burlington, MA, USA) and centrifuged at 4000× *g* in a refrigerated benchtop centrifuge (Hettich Universal 320 R, Kirchlengern, Germany) to obtain a clear supernatant. A 100-fold dilution of onion homogenate was made by adding 10 µL of the clarified onion filtrate to 0.5 mL of water in a 13 mm × 100 mm test tube. An additional 0.5 mL of water and 0.5 mL of 0.125 g L^−1^ DNPH (from Sigma-Aldrich, St. Louis, MO, USA) in 2 M HCl were added to this. The samples were placed in a 37 °C water bath (Fisherbran, MA, USA) for 10 min and 2.5 mL of 0.6 M NaOH was added. The absorbance at 515 nm was then measured (Helios Gamma, Unicam Limited, Cambridge, UK). Standards were prepared by adding 25–200 µL of 1 mM sodium pyruvate (from Sigma-Aldrich, St. Louis, MO, USA) and reducing the amount of water in the assay accordingly.

The determination of the total phenol concentration was performed with Folin–Ciocalteu reagent, according to the method by McDonadl et al. [20]. These values are expressed as gallic acid equivalents (GAE, mg 100 g^−1^ fresh weight), which is a common reference compound. Fresh onions were sliced after removing the outer skin and ends, immediately placed in liquid nitrogen, and then freeze-dried (−50 °C) for approximately 1 week, (freeze-drier LyoQuest, Telstar, Barcelona, Spain). The freeze-dried samples were placed in a freezer until ready for use. The freeze-dried onion samples were ground (grinder A10, IKA, Staufen, Germany) to a fine powder. The powder was stored under nitrogen in plastic screw-top jars in a freezer at −18 °C. Freeze-dried material (10 g) was weighed into a beaker. Methanol:water (50:50, *v*/*v*; 50 mL) was added and left for 30 min. The extract was filtered (0.45 μm, MCE Membrane Filter, Millipore, Burlington, MA, USA) and diluted (1:10, *v*/*v* with 50:50, *v*/*v* methanol:water). A diluted extract (0.5 mL of 1:10, *v*/*v*) or phenolic standard was mixed with Folin–Ciocalteu reagent (Sigma-Aldrich, St. Louis, MO, USA, 5 mL, 1:10 diluted with nanopure water) and aqueous Na_2_CO_3_ (Sigma-Aldrich, St. Louis, MO, USA, 4 mL, 1 M). Solutions were heated in a 45 °C water bath for 15 min and the total phenols were determined colorimetrically at 765 nm (Helios Gamma, Unicam Limited, Cambridge, UK). The standard curve was prepared using 0, 50, 100, 150, 200, and 250 mg L^−1^ solutions of gallic acid in methanol:water (50:50, *v*/*v*). Total phenol values are expressed as gallic acid equivalents (GAE, mg g^−1^ dry mass), which is a common reference compound.

### 2.8. RNA Extraction

Five plants per treatment, differentiating between roots, leaves, and bulbs, were frozen at −80 °C and then ground in a mortar with liquid nitrogen to obtain a fine powder. The extraction process was carried out following the manufacturer’s protocol of the NucleoSpin^®^ RNA Plant & Fungi kit (Macherey-Nagel, Düren, Germany). In this protocol, 100 mg per sample were used. Traces of contaminating DNA were removed with Thermo Scientific DNase I, RNase-free (Thermo Fisher Scientific, Waltham, MA, USA), according to the manufacturer’s protocol. The concentration and purity of the RNA were quantified with a UV/Vis NanoDrop 1000 microvolume spectrophotometer (Thermo Fischer Scientific, Waltham, MA, USA). The extracted RNA was stored at −80 °C until its use.

### 2.9. Quantitative Real-Time PCR (RT-qPCR) Analyses

The RT-qPCR of PIP1, PIP2, and TIP2 aquaporin genes from *Allium cepa* was carried out using the gene-specific primers designed by Solouki et al. [17]. According to these authors, AcTUB was used as the reference gene for the standardization of leaf and root samples, and AcACT was selected as the reference gene for normalization of bulb samples. The expression level of all the genes was measured from 2 μL of 1:10 diluted cDNA, following the system instructions in the 7500 Fast Real-Time PCR system (Applied Biosystems by Thermo Fisher Scientific, Waltham, MA, USA). The qPCR program consisted of 10 min initial denaturation at 95 °C and amplification in a two-step procedure: 10 s of denaturation at 95 °C and 40 s of annealing and extension at 60 °C for 40 cycles, followed by a dissociation stage at 50 °C for 2 min. Real-time PCR measurements were carried out in three to five independent RNA samples per treatment (biological replicates), and the Ct was determined in triplicate (technical replicates) in 96-well plates. Negative controls without cDNA were used in all the PCR reactions. Finally, the normalized expression levels were calculated using the 2^−ΔCt^ method [21].

### 2.10. Data Analysis

Statistical analyses were performed using the SPSS 29.0.0.1 software package using a one-way ANOVA. Significant differences between the values of all the parameters were determined at *p* ≤ 0.05, according to Duncan’s test. To detect outliers, the SPSS 29.0.0.1 software package was also used. The values presented are the means ± SE.

## 3. Results

The fresh weight of the plants (Table 1) revealed that there were no significant differences in leaves between the treatments and the control. However, in the bulbs, all the treatments had an effect on bulb growth, although it was only significant in the two treatments that combined foliar fertilizers and microbial root applications (FF + M and EF + M) as compared to the control, with the highest value being the one obtained in the treatment with nanoencapsulated foliar fertilization application combined with the microbial root application (EF + M). As for the roots, an increase in weight was observed in the microbial treatment alone and its combination with nanoencapsulated foliar fertilizer (M and EF + M), with this increase being significant only in the latter (EF + M).

Regarding the dry weight (Table 2), no general differences between the applied treatments with respect to the control were observed. It was only observed that the combination of nanoencapsulated foliar fertilizer and microbial root application (EF + M) showed the highest values in leaves, bulbs, and total dry weights, with a significantly higher value as compared to the non-nanoencapsulated treatment (FF + M) in leaves and total dry weight.

**Figure 1:**
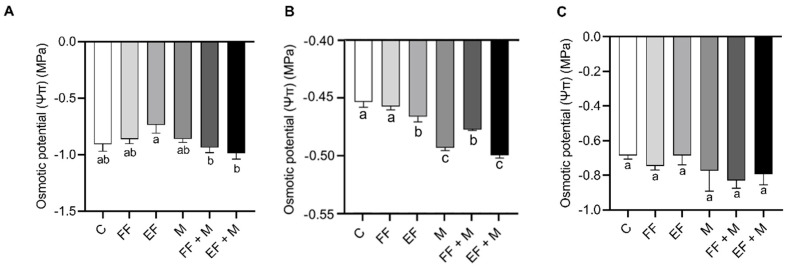
Osmotic potential (Ψπ) of onion plants (MPa). (**A**) leaves, (**B**) bulbs and (**C**) roots. Data are mean values ± standard error (SE). T (treatment), C (control), FF (free fertilizers), EF (nanoencapsulated fertilizers), M (microorganisms), FF + M (free fertilizers + microorganisms and EF + M (nanoencapsulated fertilizers + microorganisms). Different letters represent significant differences between groups after one-way ANOVA and Duncan’s test (*p* < 0.05).

The osmotic potential results showed that there was no significant change in leaves (Figure 1A) and roots (Figure 1C) in any of the treatments as compared to the control. However, in bulbs (Figure 1B), the osmotic potential significantly decreased with respect to the control after nanoencapsulated fertilization application (EF) or microorganism application (M), alone and in combination, with this decrease being higher in the M and EF + M treatments.

**Figure 2:**
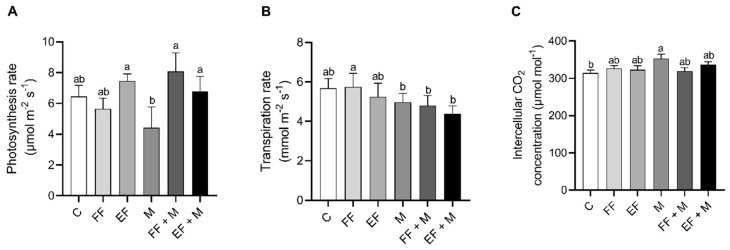
Gas exchange parameters of onion plants. (**A**) photosynthesis rate, (**B**) transpiration rate and (**C**) intercellular CO_2_ concentration. Data are mean values ± standard error (SE). T (treatment), C (control), FF (free fertilizers), EF (nanoencapsulated fertilizers), M (microorganisms), FF + M (free fertilizers + microorganisms), and EF + M (nanoencapsulated fertilizers + microorganisms). Different letters represent significant differences between groups after one-way ANOVA and Duncan’s test (*p* < 0.05).

The photosynthesis (Figure 2A) and the transpiration (Figure 2B) results in onion leaves showed that there were no significant changes between different treatments and the control group. Nevertheless, a significant increase in the intercellular CO_2_ concentration was observed in the M treatment as compared to the control (Figure 2C).

In regard to the concentration of macronutrients in onion leaves (Table 3), there were no significant differences between the treatments and the controls. In the bulbs, a higher concentration of P was measured in both foliar applications (FF and FE) and root microbial application alone (M) but not in the combined treatments (FF + M or FE + M), with respect to the control. EF also showed a significant increase in P concentration as compared to the control. In relation to potassium, the bulbs also showed a trend similar to phosphorus, although significant differences were not observed. Mg also showed significant differences, with higher values after the foliar application (FF) and microbial application (M) treatments. On the other hand, EF + M showed a significant reduction in Mg concentration.

Overall, there were no significant differences between treatments in roots. Only the reduction in S concentration was significant with respect to the control.

Regarding micronutrients (Table 4), a significant increase in Fe concentration in leaves was observed in the combination of foliar nanoencapsulated fertilizer application and the root microbial application (EF + M) as compared to the control. The EF + M treated plants also showed a significant increase in Mn as compared to control, M, and FF + M. Additionally, Zn concentrations were only significantly different in EF + M treated plants from M treated plants. The rest of the micronutrients and treatments were similar.

The foliar fertilizer application (FF) showed an increase in the micronutrient Mn, Cu, and B concentrations in bulbs as compared to control. Similarly, the microbial application (M) showed a significant increase in Zn and B. However, a significant decrease was observed in Fe with the M treatment, as compared to control, and in Zn and B in the FF + M and EF + M treatments, as compared to the treatment with only M.

Finally, in roots, only a significant reduction in Mo was observed in the FF and FF + M treatments. Additionally, significant reductions were observed between values of Fe, Mn, Cu, and Zn in M plants as compared to EF treated plants.

**Figure 3:**
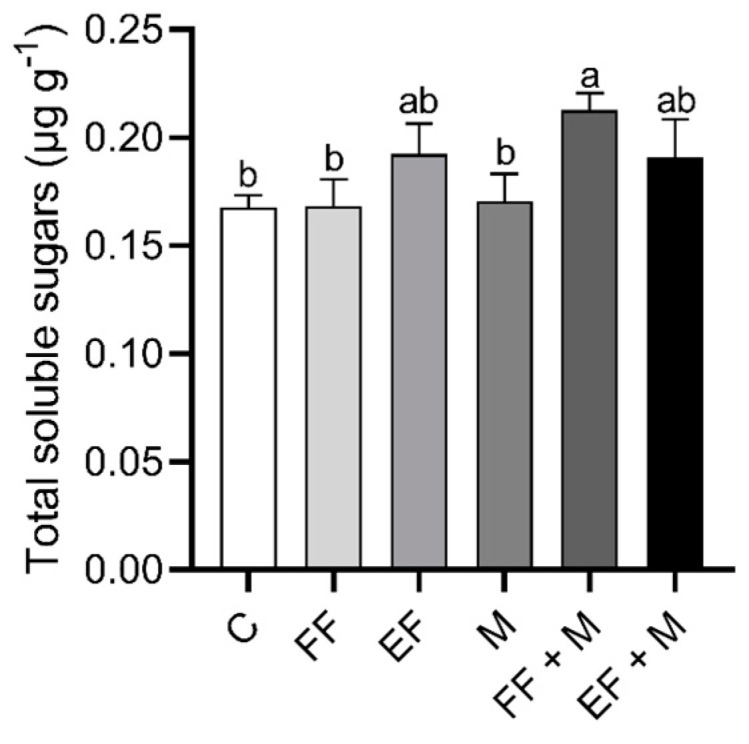
Total soluble sugar concentration in bulb (µg g^−1^). Data are mean values ± standard error (SE). T (treatment), C (control), FF (free fertilizers), EF (nanoencapsulated fertilizers), M (microorganisms), FF + M (free fertilizers + microorganisms), and EF + M (nanoencapsulated fertilizers + microorganisms). Different letters represent significant differences between groups after one-way ANOVA and Duncan’s test (*p* < 0.05).

Regarding the total content of soluble sugars in the bulb (Figure 3), an increase was observed after the foliar nanoencapsulated fertilization application (EF), alone or combined with microbial application, although it was not significant as compared to the control; the increase was significant only in the non-nanoencapsulated fertilization in combination with root microbial application (FF + M).

**Figure 4:**
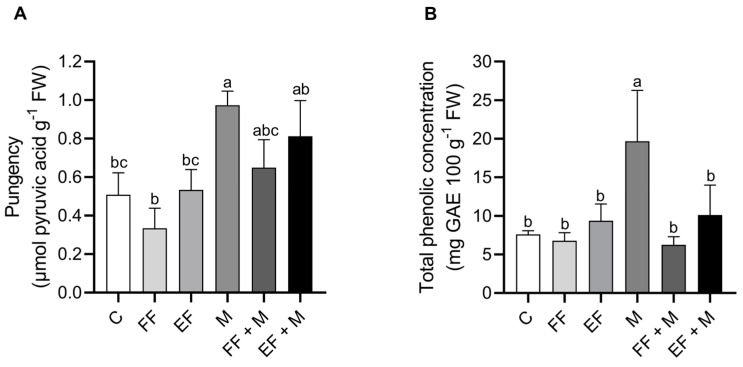
(**A**) Pungency (µmol of pyruvic acid g^−1^ FW) and (**B**) total phenolic concentration in bulb (mg GAE 100 g^−1^ FW). Data are mean values ± standard error (SE). T (treatment), C (control), FF (free fertilizers), EF (nanoencapsulated fertilizers), M (microorganisms), FF + M (free fertilizers + microorganisms), and EF + M (nanoencapsulated fertilizers + microorganisms). Different letters represent significant differences between groups after one-way ANOVA and Duncan’s test (*p* < 0.05).

The concentration of pyruvic acid (Figure 4A) and the total phenolic concentration (Figure 4B) were determined in the bulbs. Both of them were found to be significantly higher in the root microbial application (M) treatment alone. The total phenolic content did not increase in the combination treatments, although pyruvate content significantly increased in the foliar nanoencapsulated application in combination (EF + M). The application of the foliar fertilization application alone did not show any differences with respect to the control.

**Figure 5:**
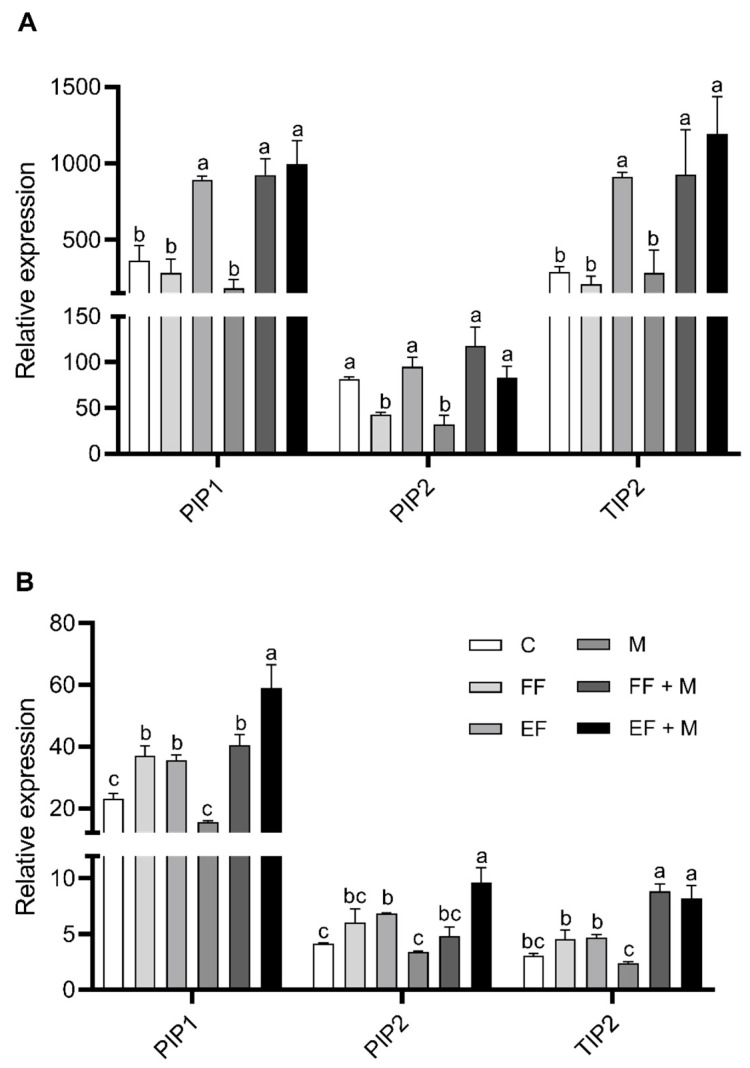
Plasma membrane intrinsic protein 1 (PIP1) and 2 (PIP2) and tonoplast intrinsic protein 2 (TIP2) relative expression in leaves (**A**) and bulbs (**B**) of onion plants. Data are mean values ± standard error (SE). T (treatment), C (control), FF (free fertilizers), EF (nanoencapsulated fertilizers), M (microorganisms), FF + M (free fertilizers + microorganisms), and EF + M (nanoencapsulated fertilizers + microorganisms). Different letters represent significant differences between groups after one-way ANOVA and Duncan’s test (*p* < 0.05).

The expression of the plasma membrane intrinsic protein 1 and 2 (PIP1 and PIP2) and the tonoplast intrinsic protein 2 (TIP2) were detected and quantified in leaves (Figure 5A) and bulbs (Figure 5B). The results from the root are not shown, as no consistent alterations were observed with the treatments.

In leaves, PIP1 and TIP2 expression showed a significant increase in the foliar nanoencapsulated fertilization application (EF) and in both root microbial applications in combination with foliar fertilization (EF + M and FF + M), but not if it was applied alone (M), in comparison to the control. In the case of PIP2, a significant decrease was observed as compared to the control with the sole application of foliar fertilization (FF) and microbial application (M), while the application of nanoencapsulated fertilization (EF) or the combination of foliar applications with microbial application (EF + M and EF + M) maintained the significance.

In bulbs, a similar trend as that from leaves was observed for PIP1 expression but, in this case, the foliar fertilizer application significantly affected its expression when it was combined with the root microbial application, as opposed to leaves. The significantly highest values were reached with the combination of foliar nanoencapsulated fertilization and microbial application (EF + M). Similar results were obtained in the case of PIP2, but only the foliar nanoencapsulated application treatments (EF and EF + M) presented significantly higher levels as compared to the control. Likewise, nanoencapsulated fertilization application plus the root microbial application (EF + M) showed the highest significance value. Lastly, with regard to TIP2 expression in bulbs, the differences were significant in the root microbial application in combination with foliar fertilization (EF + M and FF + M) with respect to the control.

## 4. Discussion

The Mediterranean soils are calcareous and usually have a low concentration of essential plant nutrients [22]. The applied microbiological treatments could also have an effect on the solubility of the nutrients in the soil, as it is well known that plants interact with the soil by emitting exudates that remain in the areas closest to the roots. These exudates could modify the communities of microorganisms that vary in quantity and composition [23]. Thus, it is not surprising that foliar micronutrient applications to the plant could modify the amount of soil microorganisms. In this way, there were only significant increases in fresh weight with the EF + M treatment. The presence of microorganism stimulated the roots’ fresh weight by almost 30%. It is known that the application of microorganisms improve root development [24,25]. However, this effect was only significant with the M + EF application, which improved not only the root fresh weight by 65% but also the weight of the bulbs by almost 50%, with respect to control plants. It has been reported that the application of fertilizers in bio-nanoencapsulated form improved not only the penetrability and absorption but also the mobilization of nutrients towards other tissues [13,14,15]. In that sense, the EF treatment was very efficient by itself, promoting the increase in bulbs by 20%. Similarly, the M treatments (alone and combined with fertilizers) increased the bulb’s fresh weight, although it was only statistically significant in the FF + M and EF + M treatments. Thus, the combination of microorganisms and fertilization treatments led to synergistic results. This is in agreement with other studies, in which the effects of the application of microorganisms were bolstered by the addition of nutrient-rich fish waste [26]. In our study, this synergy was greater when fertilizers were applied in nanoencapsulated form (EF + M), with larger production of roots and bulbs, measured as fresh weight, while root and bulb dry weighs were similar to the control in all treatments. In this regard, it must be that the increases in fresh weight detected were mainly due to water accumulation in those tissues.

Regarding the physiological parameters, some interesting results were observed in the osmotic potential of the bulbs. The osmotic potential showed that the EF and M treatments resulted in a lower water potential in the bulbs, with the lowest value observed in the M + EF treatment, which indicated an accumulation of osmolytes inside the bulb cells that favored the entry of water into them. This entry of water should increase the turgor pressure and, in consequence, cell elongation and growth. On the other hand, transpiration reached minimum values in the M treatments, pointing to water preservation promoted by the presence of microorganisms. It has been described that amending with microorganisms results in better water status regulation [27,28], which may lead to a decrease in transpiration for the same level of photosynthesis activity, depending on the ecotypes and microorganism strains used [29]. In addition, maximum values in photosynthesis rate were reached in the EF-containing treatments (EF and EF + M) and the FF + M treatment, which were correlated with an increase in total soluble carbohydrates in the onion plants. These results indicated that EF application alone was efficient in the improvement of growth, physiological parameters, and sugar production, but the combined fertilizer and microorganism amendments should result in nutrient and water accessibility, becoming optimal for enhancing onion production.

Foliar nutrient applications alone do not greatly affect plant growth or physiological parameters, although they do increase nutrient levels in both roots and bulbs. Indeed, roots from the FF and EF treatments accumulated higher levels of Fe, Mn, Cu, and Zn, but the EF treatment improved the root content of all the micronutrients, with the maximum values also observed for Mo and Zn, pointing to a greater mobilization towards the roots of the applied fertilizer. This is in agreement with the higher penetration and mobilization promoted by nanoencapsulated application [15]. Mo was clearly scarce in plants in all the treatments, being especially low in FF roots (with and without M), while, in roots from the EF treatments, it does not change. In this sense, it could be possible that the EF application affected Mo translocation in a similar manner as that described for Fe and B [14], enhancing the internal transport once the element was absorbed by the plant. The bulbs, in the case of FF, had a similar weight as the bulbs from the control plants but with a higher nutrient content (Mg, P, Mn, Cu, and B). In the EF treatment, on its part, the application of nutrients translated into a greater growth of the bulbs pointing to a greater mobilization of water and nutrients through the sink organ [30]. The dilution effect would explain why they were only significant in the case of the FF treatment, although nutrient increases were detected in the EF treatment.

Regarding the M treatment, the levels of almost all of the micronutrients in roots were lowest under this treatment. Saia et al. [31] conducted a study with fungi-based biostimulants in lettuce plants and concluded that the biostimulant effect was due to the biosynthesis of secondary compounds rather than nutrient uptake, being independent of water availability. This could explain why an accumulation of polyphenols and pungency levels in the bulbs of those plants was observed. At the same time, the plants in this treatment, on the one hand, increased their root system, which, in addition to a greater accessibility to nutrients in the soil soluble fraction, points to a greater absorption of nutrients by the roots. The contradiction could be explained by a promotion in the mobilization of nutrients toward resistance organs, such as bulbs, where an accumulation of some macro and micronutrients could be observed in those plants. However, when fertilizers were applied in combination with M (FF + M and EF + M treatments), the accumulation of nutrients in bulbs was similar to the control, although the greater fresh weight of the bulbs and the low osmotic potential pointed to an increase in water uptake in the sink organ, which could have a dilution effect in nutrient accumulation. The EF + M treatment showed a marked increase in Fe, Mn, and Zn in leaf tissues, which point to a greater accumulation of those micronutrients that was improved by the method of application. In this way, the results with the nanoencapsulated form are in agreement with previous studies in which the effectiveness, accumulation, and distribution of Zn and Fe were greater when these elements were applied in a nanoencapsulated form rather than in a non-nanoencapsulated form [13,14].

Regarding aquaporin gene expression levels, on leaves, the treatments with foliar nutrients had a different effect depending on the application format. Although the FF treatment did not change and even downregulated PIP2 aquaporin expression levels, the EF treatment upregulated the aquaporins analyzed, PIP1 and TIP2. It is well known that aquaporins accumulate strongly in tissues and cells where increased membrane transport of water and solutes is needed. In this sense, aquaporins regulate the exchange of nutrients and solutes between cellular compartments, the cytoplasm and apoplast, playing a fundamental role in osmoregulation and detoxification, as well as in the regulation of their storage and redistribution to other parts of the plant [32]. The greater penetrability of nutrients caused by its application in nanoencapsulated form, which promote the penetration of nutrients through the cuticle, reaching the abaxial epidermis and the spongy mesophyll [15], makes it necessary to deal with higher uptake of nutrients that have to be stored, regulated, and mobilized. Under these circumstances, in addition to their role as transporters of specific nutrients, aquaporins play critical functions due to their ability to transport both water and nutrients, which makes them a central element that controls the movement of water and the solutes diluted within it, through all plant tissues. Beyond the direct capacity of aquaporins to transport water and certain solutes, such as CO_2_, hydrogen peroxide, urea, ammonium, boron, or silicon, the correlation between the transport of nutrients and aquaporins is becoming evident thanks to the advances in molecular biology [33]. An example is the work by Yue et al. [34] in which the clear interrelation between nutrient transporters and maize aquaporins in leaves is highlighted. In this work, a relationship was found between PIP1 and TIP2 aquaporins and the transport of nutrients such as S, P, Mg^2+^, Fe^2+^, K^+^, Co^2+^, Cu^2+^, Cl^−^, or sugars. In addition, many PIPs and TIPs isoforms have been located surrounding the vascular bundles in leaves, and their possible contribution to the transport exchange between mesophyll and vascular tissues has been described in some plants [35]. The presence of higher levels of aquaporin expression in EF leaves as compared to plants from the FF and control treatments suggests that this is, indeed, their primary function in leaves in the EF treatments. In a similar direction, treatments with the combination of microorganism amendments and foliar fertilization increased PIP1 and TIP2 gene expression levels, pointing to similar roles in the accumulation and mobilization of nutrients, not only in leaves but also in bulb tissues [36].

In bulbs, both foliar nutrient treatments increased PIP1 and TIP2 gene expression, with and without microorganism amendments, indicating their functions in the regulation of water and/or nutrient transport to the sink organ when enough nutrients are available. However, in the EF treatments, an increase in PIP2 was observed in addition to PIP1. It is known that PIPs1 and PIPs2 interact physically. This interaction was necessary for PIP1 to reach its final position in plasma membranes [37]. Furthermore, it has been shown that the coexpression of PIPs1 and PIPs2 improves water transport through membranes, which is implemented with respect to their isolated expression [38]. These results indicate that the increase in PIP2 expression in treatments containing EF (EF and EF + M) helps to implement the effects of the high expression of PIP1 to stimulate water transport towards the bulb cells.

## 5. Conclusions

The foliar application of nutrients via nanoencapsulation technology was very effective in the penetration and mobilization of the nutrients applied and also promoting growth to a greater extent than free fertilizers. Amendments with microorganisms by themselves were not able to promote growth, although they had a strong influence on the secondary metabolites in bulbs [36]. However, the combination of both treatments (EF and M) had synergistic effects, increasing the growth of the shoots, providing higher mineral content and improving the expression of the aquaporin genes PIP1 and TIP2 in leaves. Therefore, aquaporins should lead to the mobilization of nutrients from leaves to sink organs, as bulb organs presented an overexpression of PIP2 aquaporins in combination to PIP1 and TIP2, enhancing water uptake and cell growth. Therefore, the microorganism amendments applied to onion cultivation soils should have an effect on the mineral nutrients, which could be taken up by plants. This was strongly achieved by the application of nanoencapsulated nutrients, as it increased penetrability into the leaf tissues. Furthermore, the combination EF and M was optimal for improving onion productivity via the regulation of aquaporins under field conditions.

## Figures and Tables

**Table 1:** Total fresh weight of onion plants (g plant^−1^).

T	Leaves	Bulbs	Roots	Total
C	152.22 ± 5.42 ^a^	97.68 ± 4.59 ^c^	4.55 ± 0.53 ^b^	247.27 ± 9.48 ^b^
FF	170.08 ± 10.18 ^a^	98.75 ± 12.98 ^bc^	4.82 ± 0.67 ^b^	273.50 ± 21.02 ^ab^
EF	169.08 ± 9.44 ^a^	116.33 ± 11.86 ^abc^	4.67 ± 0.59 ^b^	289.33 ± 15.57 ^ab^
M	156.12 ± 4.13 ^a^	119.80 ± 3.60 ^abc^	5.75 ± 0.88 ^ab^	285.62 ± 6.14 ^ab^
FF + M	137.50 ± 7.88 ^a^	129.92 ± 12.75 ^ab^	4.33 ± 0.71 ^b^	270.17 ± 17.84 ^ab^
EF + M	161.67 ± 12.32 ^a^	145.08 ± 17.27 ^a^	7.50 ± 0.60 ^a^	311.42 ± 15.57 ^a^

Data are mean values (n = 10) ± standard error (SE). T (treatment), C (control), FF (free fertilizers), EF (nanoencapsulated fertilizers), M (microorganisms), FF + M (free fertilizers + microorganisms), and EF + M (nanoencapsulated fertilizers + microorganisms). Different letters represent significant differences between groups after one-way ANOVA and Duncan’s test (*p* < 0.05).

**Table 2:** Total dry weight of onion plants (g plant^−1^).

T	Leaves	Bulbs	Roots	Total
C	11.62 ± 1.76 ^ab^	12.26 ± 1.45 ^a^	0.29 ± 0.11 ^a^	24.17 ± 2.87 ^ab^
FF	11.53 ± 1.44 ^ab^	9.29 ± 1.52 ^a^	0.55 ± 0.05 ^a^	21.37 ± 2.96 ^b^
EF	12.10 ± 0.15 ^ab^	12.38 ± 2.75 ^a^	0.43 ± 0.10 ^a^	24.91 ± 2.77 ^ab^
M	13.21 ± 1.19 ^ab^	12.61 ± 2.05 ^a^	0.60 ± 0.20 ^a^	26.42 ± 0.76 ^ab^
FF + M	9.73 ± 1.27 ^b^	11.43 ± 1.18 ^a^	0.26 ± 0.03 ^a^	21.42 ± 2.31 ^b^
EF + M	14.92 ± 1.86 ^a^	15.37 ± 1.88 ^a^	0.39 ± 0.14 ^a^	30.68 ± 3.22 ^a^

Data are mean values (n = 10) ± standard error (SE). T (treatment), C (control), FF (free fertilizers), EF (nanoencapsulated fertilizers), M (microorganisms), FF + M (free fertilizers + microorganisms), and EF + M (nanoencapsulated fertilizers + microorganisms). Different letters represent significant differences between groups after one-way ANOVA and Duncan’s test (*p* < 0.05).

**Table 3:** Concentration of macronutrients (g Kg^−1^ DW) in onion leaves, bulbs, and roots.

Leaves					
Treatments	Ca	K	Mg	P	S
C	17.11 ± 1.38 ^a^	25.51 ± 0.42 ^a^	2.10 ± 0.22 ^a^	1.92 ± 0.06 ^a^	5.14 ± 0.32 ^a^
FF	17.82 ± 1.52 ^a^	27.16 ± 1.04 ^a^	2.40 ± 0.19 ^a^	2.42 ± 0.10 ^a^	5.07 ± 0.26 ^a^
EF	17.24 ± 2.50 ^a^	25.61 ± 0.38 ^a^	2.14 ± 0.24 ^a^	2.28 ± 0.22 ^a^	5.12 ± 0.19 ^a^
M	17.48 ± 0.49 ^a^	27.22 ± 1.49 ^a^	2.06 ± 0.03 ^a^	2.06 ± 0.30 ^a^	5.34 ± 0.70 ^a^
FF + M	16.93 ± 0.83 ^a^	27.48 ± 2.78 ^a^	2.23 ± 0.09 ^a^	1.95 ± 0.11 ^a^	4.89 ± 0.29 ^a^
EF + M	21.86 ± 1.69 ^a^	25.92 ± 0.67 ^a^	2.48 ± 0.24 ^a^	2.09 ± 0.14 ^a^	5.13 ± 0.07 ^a^
Bulbs					
C	7.77 ± 0.32 ^a^	12.37 ± 0.38 ^ab^	1.00 ± 0.04 ^b^	2.55 ± 0.10 ^b^	3.09 ± 0.17 ^ab^
FF	9.22 ± 0.67 ^a^	13.08 ± 0.87 ^a^	1.17 ± 0.03 ^a^	2.95 ± 0.12 ^a^	3.01 ± 0.24 ^b^
EF	7.62 ± 0.38 ^a^	13.36 ± 0.99 ^a^	1.02 ± 0.05 ^b^	3.00 ± 0.10 ^a^	3.30 ± 0.29 ^ab^
M	7.94 ± 1.29 ^a^	13.89 ± 0.79 ^a^	1.19 ± 0.06 ^a^	3.10 ± 0.15 ^a^	3.78 ± 0.24 ^a^
FF + M	6.94 ± 1.04 ^a^	11.86 ± 0.49 ^ab^	0.91 ± 0.04 ^bc^	2.45 ± 0.06 ^b^	3.15 ± 0.20 ^ab^
EF + M	6.77 ± 0.22 ^a^	10.62 ± 0.17 ^b^	0.85 ± 0.02 ^c^	2.36 ± 0.11 ^b^	2.98 ± 0.19 ^b^
Roots					
C	25.87 ± 3.73 ^ab^	24.45 ± 1.21 ^ab^	4.00 ± 0.38 ^ab^	1.64 ± 0.15 ^a^	8.30 ± 0.64 ^a^
FF	27.03 ± 3.43 ^ab^	21.32 ± 1.08 ^b^	4.27 ± 0.16 ^ab^	1.51 ± 0.03 ^a^	6.31 ± 0.25 ^b^
EF	33.03 ± 3.57 ^a^	23.60 ± 2.61 ^ab^	4.65 ± 0.27 ^a^	1.66 ± 0.06 ^a^	8.52 ± 0.13 ^a^
M	21.02 ± 4.36 ^b^	28.49 ± 1.78 ^a^	3.80 ± 0.24 ^b^	1.69 ± 0.13 ^a^	7.67 ± 0.53 ^a^
FF + M	22.94 ± 2.71 ^ab^	24.82 ± 1.41 ^ab^	3.77 ± 0.06 ^b^	1.54 ± 0.22 ^a^	7.89 ± 0.41 ^a^
EF + M	24.09 ± 1.22 ^ab^	25.65 ± 3.38 ^ab^	3.90 ± 0.19 ^ab^	1.54 ± 0.03 ^a^	8.05 ± 0.41 ^a^

Data are mean values (n = 5) ± standard error (SE). T (treatment), C (control), FF (free fertilizers), EF (nanoencapsulated fertilizers), M (microorganisms), FF + M (free fertilizers + microorganisms), and EF + M (nanoencapsulated fertilizers + microorganisms). Different letters represent significant differences between groups after one-way ANOVA and Duncan’s test (*p* < 0.05).

**Table 4:** Concentration of micronutrients (mg Kg^−1^ DW) in onion leaves, bulbs, and roots.

Leaves						
Treatments	Fe	Mn	Cu	Mo	Zn	B
C	224.15 ± 60.74 ^b^	55.81 ± 2.96 ^c^	69.73 ± 15.24 ^a^	1.10 ± 0.14 ^a^	13.71 ± 0.16 ^ab^	22.18 ± 1.40 ^a^
FF	257.60 ± 44.64 ^b^	62.82 ± 3.03 ^ab^	94.15 ± 6.17 ^a^	0.93 ± 0.10 ^a^	16.02 ± 0.68 ^ab^	26.87 ± 1.02 ^a^
EF	301.90 ± 96.22 ^b^	62.80 ± 3.21 ^ab^	82.46 ± 10.51 ^a^	0.84 ± 0.11 ^a^	16.18 ± 0.21 ^ab^	25.34 ± 3.04 ^a^
M	274.59 ± 62.07 ^b^	44.09 ± 1.47 ^c^	82.16 ± 3.80 ^a^	0.77 ± 0.08 ^a^	12.91 ± 1.94 ^b^	21.45 ± 3.04 ^a^
FF + M	155.96 ± 21.71 ^b^	53.30 ± 2.07 ^b^	88.80 ± 5.13 ^a^	0.80 ± 0.06 ^a^	14.21 ± 0.79 ^ab^	20.49 ± 0.77 ^a^
EF + M	593.49 ± 160.04 ^a^	70.90 ± 2.22 ^a^	93.96 ± 11.98 ^a^	0.90 ± 0.10 ^a^	16.59 ± 1.1 ^a^	24.41 ± 3.60 ^a^
Bulbs						
C	225.25 ± 87.35 ^a^	24.25 ± 2.24 ^b^	9.86 ± 0.92 ^b^	0.42 ± 0.05 ^ab^	22.63 ± 0.69 ^bc^	18.87 ± 1.12 ^b^
FF	179.27 ± 22.65 ^a^	29.78 ± 1.31 ^a^	27.90 ± 6.62 ^a^	0.41 ± 0.01 ^ab^	25.28 ± 0.56 ^abc^	22.42 ± 1.52 ^a^
EF	179.67 ± 30.26 ^a^	25.72 ± 1.39 ^ab^	15.51 ± 0.54 ^b^	0.27 ± 0.04 ^b^	28.12 ± 2.46 ^ab^	21.56 ± 0.96 ^ab^
M	85.61 ± 11.05 ^b^	22.72 ± 1.82 ^b^	9.63 ± 1.82 ^b^	0.93 ± 0.45 ^a^	30.52 ± 4.74 ^a^	22.19 ± 0.84 ^a^
FF + M	113.65 ± 25.83 ^a^	20.87 ± 1.33 ^b^	15.10 ± 4.62 ^b^	0.26 ± 0.05 ^b^	19.47 ± 0.85 ^c^	18.51 ± 0.64 ^b^
EF + M	119.30 ± 19.20 ^a^	20.68 ± 0.82 ^b^	11.94 ± 1.43 ^b^	0.33 ± 0.06 ^ab^	19.76 ± 1.24 ^c^	18.34 ± 0.29 ^b^
Roots						
C	3115.53 ± 714.22 ^abc^	102.38 ± 9.66 ^ab^	49.14 ± 4.15 ^ab^	2.23 ± 0.49 ^a^	44.08 ± 2.15 ^ab^	24.37 ± 0.31 ^a^
FF	4019.64 ± 383.20 ^ab^	111.10 ± 8.87 ^ab^	60.08 ± 3.86 ^a^	1.20 ± 0.13 ^b^	52.93 ± 4.12 ^a^	24.28 ± 1.72 ^a^
EF	4345.51 ± 410.98 ^a^	121.30 ± 5.57 ^a^	61.02 ± 4.37 ^a^	1.76 ± 0.09 ^ab^	51.35 ± 4.01 ^a^	26.76 ± 0.59 ^a^
M	2497.82 ± 325.32 ^c^	86.84 ± 7.85 ^b^	41.78 ± 3.62 ^b^	1.72 ± 0.38 ^ab^	36.30 ± 1.36 ^b^	23.62 ± 1.42 ^a^
FF + M	2843.85 ± 140.45 ^bc^	101.02 ± 8.86 ^ab^	54.20 ± 6.40 ^ab^	1.21 ± 0.20 ^b^	45.26 ± 8.41 ^ab^	23.34 ± 2.02 ^a^
EF + M	3291.51 ± 218.69 ^abc^	97.69 ± 0.88 ^ab^	49.70 ± 4.00 ^ab^	1.53 ± 0.25 ^ab^	39.09 ± 2.89 ^ab^	22.85 ± 0.80 ^a^

Data are mean values (n = 5) ± standard error (SE). T (treatment), C (control), FF (free fertilizers), EF (nanoencapsulated fertilizers), M (microorganisms), FF + M (free fertilizers + microorganisms), and EF + M (nanoencapsulated fertilizers + microorganisms). Different letters represent significant differences between groups after one-way ANOVA and Duncan’s test (*p* < 0.05).

## Data Availability

Data are contained within the article can be available upon request.

## References

[1] Gautam A., Pandey A.K. (2021). Aquaporins Responses under Challenging Environmental Conditions and Abiotic Stress Tolerance in Plants. Bot. Rev..

[2] Food and Agriculture Organization of the United Nations (FAO) (2021). World Food and Agriculture—Statistical Yearbook 2021.

[3] Brewster J.L., Atherton J., Rees A. (2008). Onions and other vegetable Alliums. Crop Production Science in Horticulture Series.

[4] Pascale A., Proietti S., Pantelides I.S., Stringlis I.A. (2020). Modulation of the Root Microbiome by Plant Molecules: The Basis for Targeted Disease Suppression and Plant Growth Promotion. Front. Plant Sci..

[5] Sofo A., Elshafie H.S., Camele I. (2020). Structural and Functional Organization of the Root System: A Comparative Study on Five Plant Species. Plants.

[6] Kumar S., Diksha, Sindhu S.S., Kumar R. (2022). Biofertilizers: An Ecofriendly Technology for Nutrient Recycling and Environmental Sustainability. Curr. Res. Microb. Sci..

[7] Hakim S., Naqqash T., Nawaz M.S., Laraib I., Siddique M.J., Zia R., Mirza M.S., Imran A. (2021). Rhizosphere Engineering with Plant Growth-Promoting Microorganisms for Agriculture and Ecological Sustainability. Front. Sustain. Food Syst..

[8] Mącik M., Gryta A., Frąc M. (2020). Biofertilizers in Agriculture: An Overview on Concepts, Strategies and Effects on Soil Microorganisms. Advances in Agronomy.

[9] Abd-Ellatif S., Ibrahim A.A., Safhi F.A., Abdel Razik E.S., Kabeil S.S.A., Aloufi S., Alyamani A.A., Basuoni M.M., ALshamrani S.M., Elshafie H.S. (2022). Green Synthesized of *Thymus vulgaris* Chitosan Nanoparticles Induce Relative WRKY-Genes Expression in *Solanum lycopersicum* against *Fusarium solani*, the Causal Agent of Root Rot Disease. Plants.

[10] Aqeel U., Aftab T., Khan M.M.A., Naeem M., Khan M.N. (2022). A Comprehensive Review of Impacts of Diverse Nanoparticles on Growth, Development and Physiological Adjustments in Plants under Changing Environment. Chemosphere.

[11] Begum P., Ikhtiari R., Fugetsu B. (2011). Graphene Phytotoxicity in the Seedling Stage of Cabbage, Tomato, Red Spinach, and Lettuce. Carbon. N. Y..

[12] Safiuddin M., Gonzalez M., Cao J., Tighe S.L. (2014). State-of-the-Art Report on Use of Nano-Materials in Concrete. Int. J. Pavement Eng..

[13] Ríos J.J., García-Ibáñez P., Carvajal M. (2019). The Use of Biovesicles to Improve the Efficiency of Zn Foliar Fertilization. Colloids Surf. B Biointerfaces.

[14] Ríos J.J., Yepes-Molina L., Martínez-Alonso A., Carvajal M. (2020). Nanobiofertilization as a Novel Technology for Highly Efficient Foliar Application of Fe and B in Almond Trees: Nanobiofertilization in Almond Trees. R. Soc. Open Sci..

[15] Ríos J.J., López-Zaplana A., Bárzana G., Martínez-Alonso A., Carvajal M. (2021). Foliar Application of Boron Nanoencapsulated in Almond Trees Allows B Movement Within Tree and Implements Water Uptake and Transport Involving Aquaporins. Front. Plant Sci..

[16] Chaumont F., Tyerman S. (2017). Plant Aquaporins from Transport to Signaling.

[17] Solouki A., Berna-Sicilia J.Á., Martínez-Alonso A., Ortíz-Delvasto N., Bárzana G., Carvajal M. (2023). Onion Plants (*Allium cepa* L.) React Differently to Salinity Levels According to the Regulation of Aquaporins. Heliyon.

[18] Yemm E.W., Willis A.J. (1954). The Estimation of Carbohydrates in Plant Extracts by Anthrone.

[19] Anthon G.E., Barrett D.M. (2003). Modified Method for the Determination of Pyruvic Acid with Dinitrophenylhydrazine in the Assessment of Onion Pungency. J. Sci. Food Agric..

[20] Mcdonald S., Prenzler P.D., Antolovich M., Robards K. (2000). Phenolic Content and Antioxidant Activity of Olive Extracts. Food Chem..

[21] Schmittgen T.D., Livak K.J. (2008). Analyzing Real-Time PCR Data by the Comparative CT Method. Nat. Protoc..

[22] Rashid A., Ryan J. (2004). Micronutrient Constraints to Crop Production in Soils with Mediterranean-Type Characteristics: A Review. J. Plant Nutr..

[23] Badri D.V., Vivanco J.M. (2009). Regulation and Function of Root Exudates. Plant Cell Environ..

[24] Shahrajabian M.H., Chaski C., Polyzos N., Petropoulos S.A. (2021). Biostimulants Application: A Low Input Cropping Management Tool for Sustainable Farming of Vegetables. Biomolecules.

[25] Pokluda R., Ragasová L.N., Jurica M., Kalisz A., Komorowska M., Niemiec M., Caruso G., Gąstoł M., Sekara A. (2023). The Shaping of Onion Seedlings Performance through Substrate Formulation and Co-Inoculation with Beneficial Microorganism Consortia. Front. Plant Sci..

[26] Abdelhameed R.E., Metwally R.A. (2022). Assessment of Beneficial Fungal Microorganism’s Bio-Efficacy in Stimulating Morphological and Physiological Parameters of *Allium cepa* Plants Grown in Soil Amended with Fish Wastes. BMC Plant Biol..

[27] Abeed A.H.A., Mahdy R.E., Alshehri D., Hammami I., Eissa M.A., Abdel Latef A.A.H., Mahmoud G.A.E. (2022). Induction of Resilience Strategies against Biochemical Deteriorations Prompted by Severe Cadmium Stress in Sunflower Plant When *Trichoderma* and Bacterial Inoculation Were Used as Biofertilizers. Front. Plant Sci..

[28] Almaroai Y.A., Eissa M.A. (2020). Role of Marine Algae Extracts in Water Stress Resistance of Onion Under Semiarid Conditions. J. Soil. Sci. Plant Nutr..

[29] Cabrera-Puerto R.J., Ruiz-Gómez F.J., Navarro-Cerrillo R.M. (2023). Beneficial Microorganisms and Water Stress Influence *Quercus ilex* Seedlings’ Response to Phytophthora Cinnamomi Rands. Forests.

[30] Blanco E.L., Rada F., Paolini J. (2023). The Role of a Microbial Consortium on Gas Exchange and Water Relations in *Allium cepa* L. under Water and Nutritional Deficit Conditions. Arch. Microbiol..

[31] Saia S., Colla G., Raimondi G., Di Stasio E., Cardarelli M., Bonini P., Vitaglione P., De Pascale S., Rouphael Y. (2019). An Endophytic Fungi-Based Biostimulant Modulated Lettuce Yield, Physiological and Functional Quality Responses to Both Moderate and Severe Water Limitation. Sci. Hortic..

[32] Maurel C., Verdoucq L., Luu D.T., Santoni V. (2008). Plant Aquaporins: Membrane Channels with Multiple Integrated Functions. Annu. Rev. Plant Biol..

[33] Bárzana G., Carvajal M. (2020). Genetic Regulation of Water and Nutrient Transport in Water Stress Tolerance in Roots. J. Biotechnol..

[34] Yue X., Zhao X., Fei Y., Zhang X. (2012). Correlation of Aquaporins and Transmembrane Solute Transporters Revealed by Genome-Wide Analysis in Developing Maize Leaf. Comp. Funct. Genom..

[35] Heinen R.B., Ye Q., Chaumont F. (2009). Role of Aquaporins in Leaf Physiology. J. Exp. Bot..

[36] Elshafie H.S., Camele I. (2021). An Overview of Metabolic Activity, Beneficial and Pathogenic Aspects of *Burkholderia* spp.. Metabolites.

[37] Jozefkowicz C., Rosi P., Sigaut L., Soto G., Pietrasanta L.I., Amodeo G., Alleva K. (2013). Loop A Is Critical for the Functional Interaction of Two Beta Vulgaris PIP Aquaporins. PLoS ONE.

[38] Bienert M.D., Diehn T.A., Richet N., Chaumont F., Bienert G.P. (2018). Heterotetramerization of Plant PIP1 and PIP2 Aquaporins Is an Evolutionary Ancient Feature to Guide PIP1 Plasma Membrane Localization and Function. Front. Plant Sci..

